# Longevity Code: Lipidome Associations and Mediated Metabolite Effects

**DOI:** 10.1002/brb3.70937

**Published:** 2025-11-11

**Authors:** Yunfeng Yu, Xinyu Yang, Juan Deng, Jingyi Wu, Rong Yu, Qin Xiang

**Affiliations:** ^1^ School of Traditional Chinese Medicine Hunan University of Chinese Medicine Changsha China; ^2^ The First Hospital of Hunan University of Chinese Medicine Changsha China; ^3^ The Third School of Clinical Medicine Zhejiang Chinese Medical University Hangzhou China

**Keywords:** lipidome, longevity, mediated effect, Mendelian randomization, metabolites

## Abstract

**Objective:**

The effects of lipidome on longevity and the role of metabolites in between have not been fully elucidated. The purpose of this study was to assess the causal effects of lipidome on longevity and the mediated effects of metabolites using Mendelian randomization (MR).

**Methods:**

Data on lipidome, metabolites, and longevity were acquired in the genome‐wide association studies, and single‐nucleotide polymorphisms meeting the basic assumptions of MR were selected. Subsequently, inverse variance weighted was employed as the primary method to analyze the causal effects of lipidome on longevity and the mediated effects of metabolites. Finally, MR‐Egger intercept was used to assess horizontal pleiotropy in the results. Cochran's *Q* and leave‐one‐out sensitivity analysis were used to assess the heterogeneity and robustness of the results, respectively.

**Results:**

The MR analysis showed that sterol ester (27:1/18:3) decreased genetic susceptibility to longevity (90th) by reducing ethylenediaminetetraacetic acid (EDTA) levels (mediated proportion: 13.60%; mediated effect: −0.020, 95% confidence interval [CI] −0.039 to −0.002, *p* = 0.033); triacylglycerol (52:2) decreased genetic susceptibility to longevity (90th) by reducing EDTA levels (mediated proportion: 11.20%; mediated effect: −0.025, 95% CI −0.045 to −0.004, *p* = 0.020); triacylglycerol (54:5) decreased genetic susceptibility to longevity (90th) by increasing 1‐arachidonoyl‐gpc (20:4n6) levels (mediated proportion:8.37%; mediated effect: −0.010, 95% CI −0.021 to −9.44e‐05, *p* = 0.048); phosphatidylcholine (O‐16:0_18:2) increased genetic susceptibility to longevity (90th) by increasing EDTA levels (mediated proportion: 22.10%; mediated effect: 0.029, 95% CI 0.007–0.050, *p* = 0.008). MR‐Egger intercept showed that these results lacked horizontal pleiotropy (*p* ≥ 0.05). Cochran's *Q* and sensitivity analysis showed that the MR results had no heterogeneity and were robust.

**Conclusion:**

The MR analysis revealed four pathways through which lipidome regulates longevity via metabolites. Lipidome such as sterol ester (27:1/18:3), phosphatidylcholine (O‐16:0_18:2), triacylglycerol (52:2), and triacylglycerol (54:5), as well as metabolites such as EDTA and 1‐arachidonoyl‐gpc (20:4n6), may play important roles in longevity.

## Graphical abstract

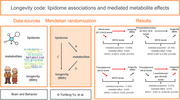

**Abbreviations**
AD: Alzheimer's disease
CI: confidence interval
EDTA: ethylenediaminetetraacetic acid
GWAS: genome‐wide association studies
MR: Mendelian randomization
OR: odds ratio
SNP: single‐nucleotide polymorphism
SOD: superoxide dismutase

## 1 Introduction

Longevity has long been one of the concerns of human society (The Lancet Healthy Longevity 2020). With the continuous improvement of medical technology and living standards, people's pursuit of longevity has become increasingly strong (Olshansky 2022; P. Liu et al. 2025). Since the last century, the average life expectancy of human beings worldwide has increased from 30 years in 1900 to 65 years in 2000 (Oeppen and Vaupel 2002). According to the World Health Organization, by 2050, the number of people over the age of 60 will increase to 2.1 billion, and the number of people over the age of 80 will reach 426 million (Austad and Fischer 2016). The continued increase in average life expectancy suggests that longevity has a degree of flexibility and adjustability. Usually, we refer to the duration of life up to a particular age of 90 years or more as longevity (Murabito et al. 2012). Longevity does not only imply an increase in age but also a state of health, vitality, and quality of life that is still maintained at an advanced age. With the accelerating aging of the world's population, it is particularly significant to explore the underlying mechanisms of longevity to slow down aging and increase longevity. Early genome‐wide association studies (GWAS) revealed that human longevity is influenced by genetic factors (J. Deelen et al. 2019). However, recent studies have found that, regardless of twin or pedigree study design, estimates of the heritability of longevity for the entire population range from 0.01 to 0.27 (van den Berg et al. 2017), which implies that environmental factors have a pivotal influence on human longevity.

Lipidome refers to the total number of lipid molecules in an organism, which is closely related to the health and diseases of the organism. It is primarily divided into fatty acids, phospholipids (glycerophospholipids and sphingolipids), and neutral lipids (triglycerides and cholesterol/sterol lipids), which regulate aging and longevity through multiple metabolic pathways (Mutlu et al. 2021, Meng et al. 2023). A previous observational study showed that lipids are strongly associated with an increased risk of all‐cause mortality and reduced odds of living a long life (Wang et al. 2023). However, due to limited evidence, it is not sufficient to fully explain the impact of lipidome and its metabolites on longevity. Additionally, due to the predominantly cross‐sectional nature of previous studies, they unavoidably suffer from the influence of reverse causation and confounding factors. Therefore, there is an urgent need for an effective and comprehensive approach to assess the effects of lipidome‐regulated metabolites on longevity, which will provide ideas for the unraveling of longevity mechanisms and the drugs development.

Mendelian randomization (MR) is an epidemiological analysis method that assesses the causal effects between two factors through genetic variants (Bowden and Holmes 2019). Because MR is based on Mendel's laws of segregation, it avoids the effects of reverse causation and measurement error and has a higher degree of confidence in causal inferences than traditional study designs (Emdin et al. 2017). In this MR study, we assessed the causal relationships between lipidome and metabolites, metabolites and longevity, as well as lipidome and longevity, and elucidated the pathways through which lipidome regulates longevity via metabolites.

## 2 Materials and Methods

### 2.1 Study Design

**FIGURE 1:**
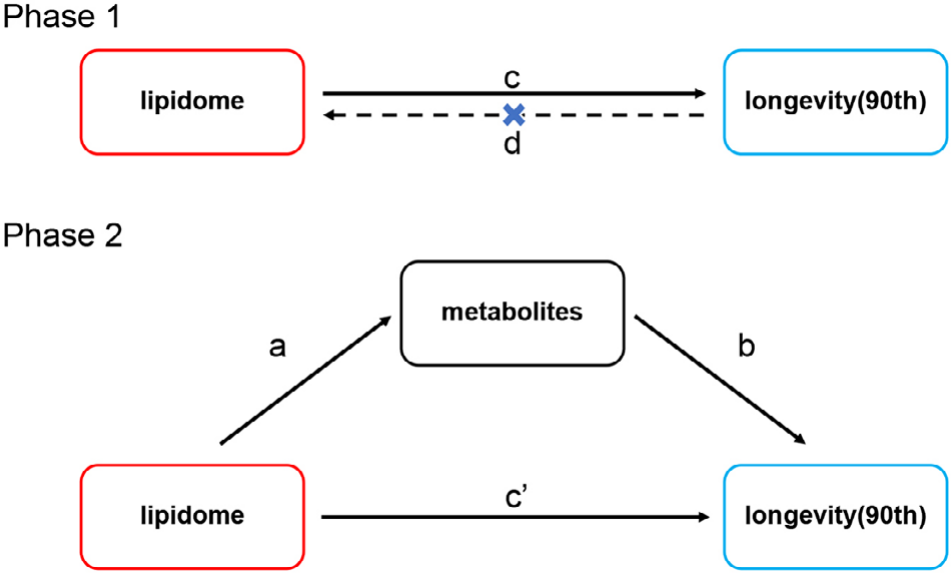
MR design diagrams of “lipidome—metabolites—longevity (90th).” The *a* is the effect of lipidome on metabolites; the *b* is the effect of metabolites on longevity (90th); the *c* is the total effect of lipidome on longevity (90th); the *c*' is the direct effect of lipidome on longevity (90th); and the *d* is the total effect of longevity (90th) on lipidome. The algorithm for the mediated effect is *a* × *b*. The algorithm for the mediated proportion is (*a* × *b*)/*c*.

This MR study was reported according to the Strengthening the Reporting of Observational Studies in Epidemiology Using MR guidelines (Skrivankova et al. 2021). It consisted of two phases; see Figure 1. Phase 1: Assessed the causal relationship between lipidome and longevity using a bidirectional MR study. It required a significant causal effect of lipidome on longevity but no significant effect of longevity on lipidome. Phase 2: Assessed the mediated effects of metabolites in the impact of lipidome on longevity. It required a significant causal effect of lipidome on metabolites and a significant causal effect of metabolites on longevity.

### 2.2 Data Sources

Data for lipidome were derived from the GeneRISK cohort (Ottensmann et al. 2023). The cohort recruited 7,174 aged 45–66 years in southern Finland between 2015 and 2017. It randomly invited 4,857 individuals through population registers and recruited 1,369 individuals and 1,116 blood donors through private occupational healthcare organizations and advertisements, respectively. Of these, individuals under guardianship, those with a history of atherosclerotic cardiovascular disease, and pregnant women were excluded from the study. Prior to the collection of blood samples, participants were asked to fast overnight for 10 h. Subsequently, participants' blood samples were collected for plasma, serum, and DNA extractions and ultimately compiled into a GWAS of 179 lipid species in 13 lipid classes.

Data for metabolites were obtained from the Canadian Longitudinal Study on Aging cohort (Y. Chen, Lu, et al. 2023). The cohort followed > 50,000 participants aged 45 to 85 years and ultimately included 8,299 unrelated European subjects. After collecting plasma samples from the participants, MetabolonHD4 was used to analyze the plasma samples for 1458 metabolites, ultimately compiling 1,091 metabolites and 309 metabolite ratios for GWAS.

Data for longevity (90th), numbered GCST008598, were obtained from the study conducted by J. Deelen et al. (2019), which included 20 European ancestry cohorts and 36,745 sample sizes from the United States and Europe. It included 11,262 cases surviving at or beyond the age corresponding to the 90th survival percentile and 25,483 controls. In the study, cases were individuals who lived to an age above the 90th percentile based on cohort life tables from census data from the appropriate country, sex, and birth cohort. Controls were individuals who died at or before the age at the 60th percentile or whose age at the last follow‐up visit was at or before the 60th percentile age. As these data were publicly available, no additional ethical approval was required.

### 2.3 Selection of Genetic Instrumental Variables (IVs)

To ensure the validity of the MR analysis, we adopted several stringent steps for selecting IVs while adhering to the three core MR assumptions of relevance, independence, and exclusion. Initially, we attempted to use a genome‐wide significance threshold of *p* < 5 × 10^−8^ to identify single‐nucleotide polymorphisms (SNPs), but this criterion substantially reduced the number of eligible IVs. Therefore, to retain adequate statistical power, we adopted a relaxed threshold of *p* < 5 × 10^−5^ for SNPs associated with lipidomic and metabolite traits, and a slightly more stringent threshold of *p* < 5 × 10^−6^ for longevity‐related SNPs (90th percentile survival). To ensure independence among SNPs, linkage disequilibrium (LD) pruning was performed with a window size of 10,000 kb and an *R* ^2^ < 0.001 threshold, retaining only independent variants. Instrument strength was assessed using the *F*‐statistic, calculated as *F* = (*R* ^2^/[1 − *R* ^2^]) × ([*N* − *K* − 1]/*K*), where *R* ^2^ represents the variance explained by the SNP, *N* the sample size, and *K* the number of instruments; only SNPs with *F* > 10 were retained to minimize weak instrument bias. To fulfill the independence assumption and avoid confounding, we used PhenoScanner and Google Scholar to exclude SNPs associated with potential confounders such as socioeconomic status, smoking, or comorbidities. Exposure and outcome datasets were then harmonized by aligning effect alleles, excluding ambiguous, mismatched, and duplicate SNPs based on allele frequency checks. Finally, horizontal pleiotropy was assessed using the MR‐Pleiotropy Residual Sum and Outlier (MR‐PRESSO) method, which identifies outlier SNPs that may bias causal estimates. A significant distortion test (*p* < 0.05) indicated that the original causal estimate was biased, and removing outliers led to a more reliable causal inference.

### 2.4 Data Analysis

First, two‐sample MR was conducted to analyze the bidirectional causality between lipidome and longevity (90th). It aimed to identify the lipidome with significant effects on longevity (90th) and unaffected by reverse causality and obtain the total effect of lipidome on longevity (90th) (c). Second, two‐sample MR was employed to assess the causal effects of the lipidome on metabolites (a) and the causal effects of metabolites on longevity (90th) (b), with the objective of screening lipidome and metabolites that satisfy the “lipidome—metabolites—longevity (90th)” pathway. Third, based on the obtained data, the mediated effects and mediated proportions of metabolites in lipidome‐regulated longevity (90th) were calculated. Mediated effect = a × b, with CI calculated by the delta method. Mediated proportion = (a × b)/c.

All analyses were performed using R software (version 4.3.1) with the TwoSampleMR package (version 0.5.7). The inverse variance weighted (IVW) method was set as the primary analytical approach because it provides unbiased causal estimates under the assumption of no horizontal pleiotropy. To enhance the robustness of the findings, the weighted median method, which is less sensitive to outliers, and the MR‐Egger regression, which accounts for potential pleiotropy, were applied as secondary analytical tools. Causal effect estimates were reported as odds ratios (ORs) with 95% confidence intervals (CIs). ORs quantify both the magnitude and direction of the causal effect. To assess potential directional horizontal pleiotropy, we used the MR‐Egger intercept test, which evaluates whether the intercept from the MR‐Egger regression significantly deviates from zero. A non‐zero intercept indicates that some genetic instruments may directly affect the outcome independent of the exposure, violating the exclusion restriction assumption and suggesting potential pleiotropic bias. In this study, a non‐significant intercept (*p* ≥ 0.05) was interpreted as no evidence of directional pleiotropy, thereby supporting the validity of the instrumental variables and the reliability of the causal estimates. Finally, Cochran's *Q* test and leave‐one‐out analyses were used to evaluate heterogeneity and perform sensitivity assessments, respectively. Results were considered homogeneous when *p* ≥ 0.05, and the causal estimates were deemed robust when the exclusion of any single SNP did not materially alter the combined effect sizes.

## 3 Results

### 3.1 Genetic IVs

The MR analysis reported four pathways of “lipidome—metabolites—longevity (90th).” It contained four causal effects of “lipidome—metabolites,” two causal effects of “metabolites—longevity (90th),” and four causal effects of “lipidome—longevity (90th).” The related SNPs are shown in Table S1.

### 3.2 Lipidome—Metabolites

**FIGURE 2:**
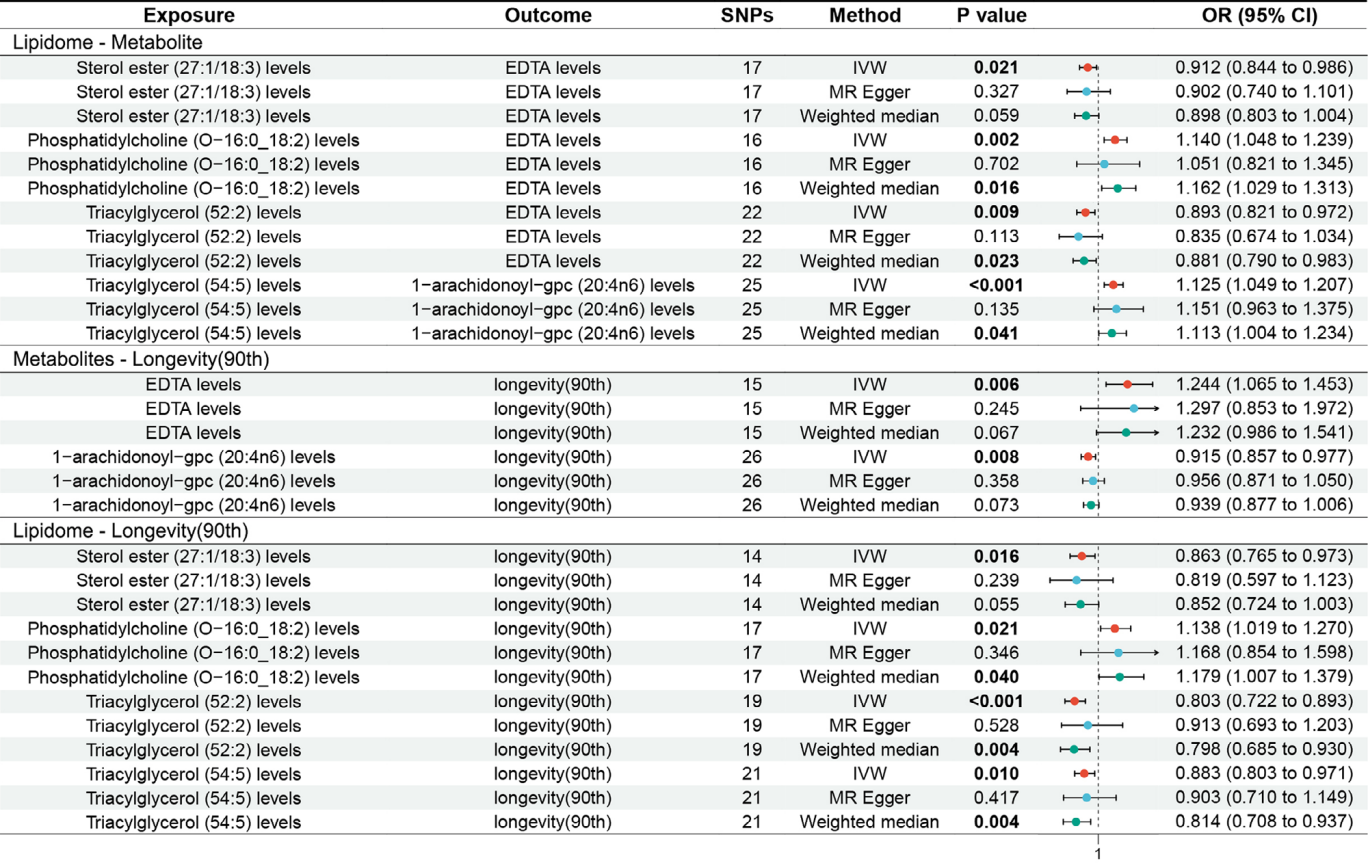
Forest plot of MR analysis for lipidome—metabolites—longevity (90th). MR, Mendelian randomization.

The MR analysis showed that sterol ester (27:1/18:3) levels were associated with a reduced genetic susceptibility to ethylenediaminetetraacetic acid (EDTA) levels (OR 0.912, 95% CI 0.844–0.986, *p* = 0.021); triacylglycerol (52:2) levels were associated with a decreased genetic susceptibility to EDTA levels (OR 0.893, 95% CI 0.821–0.972, *p* = 0.009); phosphatidylcholine (O‐16:0_18:2) levels were associated with an increased genetic susceptibility to EDTA levels (OR 1.140, 95% CI 1.048–1.239, *p* = 0.002); triacylglycerol (54:5) levels were associated with an increased genetic susceptibility to 1‐arachidonoyl‐gpc (20:4n6) levels (OR 1.125, 95% CI 1.049–1.207, *p* < 0.001), see Figure 2 for the forest plot and Figure S1 for the scatter plot. MR‐Egger showed no horizontal pleiotropy (*p* ≥ 0.05) in the results; see Table S2.

### 3.3 Metabolites—Longevity (90th)

The MR analysis showed that EDTA levels were associated with an increased genetic susceptibility to longevity (90th) (OR 1.244, 95% CI 1.065–1.453, *p* = 0.006), while 1‐arachidonoyl‐gpc (20:4n6) levels were associated with a decreased genetic susceptibility to longevity (OR 0.915, 95% CI 0.857–0.977, *p* = 0.008); see Figure 2 for the forest plot and Figure S1 for the scatter plot. MR‐Egger showed no horizontal pleiotropy in the results (*p* ≥ 0.05); see Table S2.

### 3.4 Lipidome—Longevity (90th)

The MR analysis showed that sterol ester (27:1/18:3) levels (OR 0.863, 95% CI 0.765–0.973, *p* = 0.016), triacylglycerol (52:2) levels (OR 0.803, 95% CI 0.722–0.893, *p* < 0.001), and triacylglycerol (54:5) levels (OR 0.883, 95% CI 0.803–0.971, *p* = 0.010) were associated with a reduced genetic susceptibility to longevity (90th), while phosphatidylcholine (O‐16:0_18:2) levels were associated with an increased genetic susceptibility to longevity (90th) (OR 1.138, 95% CI 1.019–1.270, *p* = 0.021); see Figure 2 for the forest plot and Figure S1 for the scatter plot. MR‐Egger showed no horizontal pleiotropy in the results (*p* ≥ 0.05); see Table S2.

### 3.5 Lipidome—Metabolites—Longevity (90th)

**FIGURE 3:**
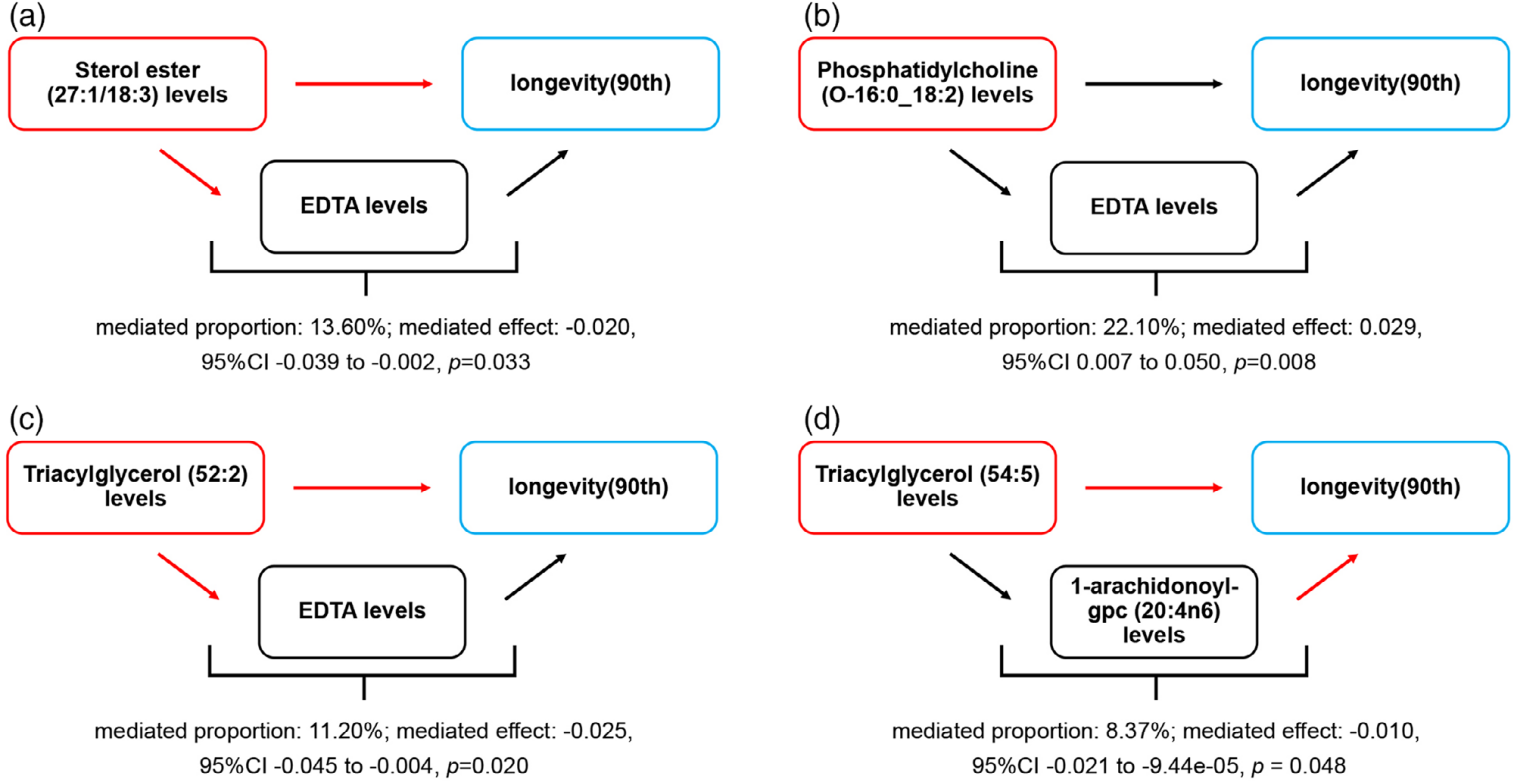
Schematic diagram of the metabolites mediated effect. (A) The mediated effect of ethylenediaminetetraacetic acid (EDTA) levels between sterol ester (27:1/18:3) levels and longevity (90th). (B) The mediated effect of EDTA levels between phosphatidylcholine (O‐16:0_18:2) levels and longevity (90th). (C) The mediated effect of EDTA levels between triacylglycerol (52:2) levels and longevity (90th). (D) The mediated effect of 1‐arachidonoyl‐gpc (20:4n6) levels between triacylglycerol (54:5) levels and longevity (90th). Black arrows indicate increases, and red arrows indicate decreases.

The MR analysis reported four pathways of “lipidome—metabolites—longevity (90th),” as shown in Figure 3.

Sterol ester (27:1/18:3) levels reduced genetic susceptibility to longevity (90th) by reducing EDTA levels. EDTA levels accounted for 13.60% of the reduction in longevity (90th) associated with sterol ester (27:1/18:3) levels (mediated proportion: 13.60%; mediated effect: −0.020, 95% CI ‐0.039 to −0.002, *p* = 0.033).

Phosphatidylcholine (O‐16:0_18:2) levels increased genetic susceptibility to longevity (90th) by increasing EDTA levels. EDTA levels accounted for 22.10% of the reduction in longevity (90th) associated with phosphatidylcholine (O‐16:0_18:2) level (mediated proportion: 22.10%; mediated effect: 0.029, 95% CI 0.007–0.050, *p* = 0.008).

Triacylglycerol (52:2) levels reduced genetic susceptibility to longevity (90th) by reducing EDTA levels. EDTA levels accounted for 11.20% of the reduction in longevity (90th) associated with triacylglycerol (52:2) levels (mediated proportion: 11.20%; mediated effect: −0.025, 95% CI −0.045 to −0.004, *p* = 0.020).

Triacylglycerol (54:5) levels decreased genetic susceptibility to longevity (90th) by increasing 1‐arachidonoyl‐gpc (20:4n6) levels. 1‐arachidonoyl‐gpc (20:4n6) levels accounted for 8.37% of the reduction in longevity (90th) associated with triacylglycerol (54:5) levels (mediated proportion: 8.37%; mediated effect: −0.010, 95% CI −0.021 to −9.44e‐05, *p* = 0.048).

### 3.6 Heterogeneity and Sensitivity Analysis

Cochran's *Q* suggested no heterogeneity in the MR analysis results (*p* ≥ 0.05), see Figure S2 and Table S3. Sensitivity analysis showed that the MR analysis results were robust, see Figure S3.

## 4 Discussion

### 4.1 Research Background and Results

With the extension of the human lifespan and the arrival of an aging society, longevity has become one of the focuses of people's attention (The Lancet Healthy Longevity 2020). It is not only a symbol of individual physiological health and psychological well‐being but also an important support for social stability and development. The mechanism of longevity is a research hotspot in the field of life sciences, and elucidating the mechanism of longevity is the basis for developing drugs that delay aging and extend lifespan. A previous study showed that there are significant differences in the levels of certain lipidome and lipid metabolites in different age groups (Ma et al. 2023; Meng et al. 2025), pointing to the possibility that lipidome and their metabolites may be associated with aging or longevity. However, constrained by limited clinical evidence, existing studies cannot adequately explain the impact of lipidome on longevity and the mediated effect of metabolites. To the best of our knowledge, this is the first MR analysis to explore the causal effects of lipidome‐regulated metabolites on longevity. The findings showed that sterol ester (27:1/18:3) levels and triacylglycerol (52:2) levels reduced genetic susceptibility to longevity (90th) by decreasing EDTA levels, triacylglycerol (54:5) levels reduced genetic susceptibility to longevity (90th) by decreasing 1‐arachidonoyl‐gpc (20:4n6) levels, and phosphatidylcholine (O‐16:0_18:2) levels increased genetic susceptibility to longevity (90th) by increasing EDTA levels. It revealed that lipidome such as sterol ester (27:1/18:3), triacylglycerol (52:2), triacylglycerol (54:5), and Phosphatidylcholine (O‐16:0_18:2), as well as metabolites such as EDTA and 1‐arachidonoyl‐ gpc are closely linked to longevity.

### 4.2 Effects of Lipidome on Longevity

Sterol ester (27:1/18:3) refers to sterol lipid formed by combining sterols with fatty acids through ester bonds, containing a special long‐chain unsaturated fatty acid (one double bond of 27 carbon atoms) and an α‐linolenic acid (three double bonds of 18 carbon atoms). Recent studies have shown that phytosterol is a risk factor for atherosclerosis (Makhmudova et al. 2021). Cholesterol is the most common sterol (Wegner et al. 2023), whose homeostatic disorder plays a crucial role in cardiovascular and neurodegenerative diseases. In an earlier study, the Framingham study reported that serum cholesterol levels were significantly associated with an increased risk of coronary heart disease (Kannel et al. 1971). Subsequently, two large prospective cohort studies conducted in China and South Korea noted that persistently high residual cholesterol concentrations significantly increased the risk of cardiovascular disease in patients (Yi et al. 2019; Wu et al. 2023). Cardiovascular disease, as the leading cause of death worldwide, showed a significant negative correlation with life expectancy (Apostu et al. 2021). It suggests that cholesterol reduces life expectancy by increasing the risk of cardiovascular disease. Additionally, cholesterol has been found to be associated with neurodegenerative diseases such as Alzheimer's disease (AD). A previous experimental study showed that mice on a consistently high cholesterol diet developed overall features of AD, including impaired spatial memory capacity, accumulation of Aβ oligomers within neurons, reduced synaptophysin immunoreactivity, and abnormal hippocampal tau phosphorylation (Umeda et al. 2012). A subsequent study noted that statins play a potential role in preventing or treating AD by modulating cholesterol (Shepardson et al. 2011). These pieces of evidence support that cholesterol reduces human life expectancy by increasing the risk of cardiovascular and neurodegenerative diseases, pointing to sterols and sterol lipids may be risk factors for longevity.

Interestingly, α‐linolenic acid is considered to be a fatty acid with anti‐inflammatory and antioxidant properties, playing an active role in diseases of the nervous system, cardiovascular system, and osteoporosis (K. B. Kim et al. 2014). A related meta‐analysis showed a 10% reduction in the risk of death from coronary heart disease (RR: 0.90; 95% CI: 0.83–0.99) for each 1 g/day increase in dietary intake of α‐linolenic acid (Pan et al. 2012). Additionally, each 0.5% increase in α‐linolenic acid concentration in adipose tissue was associated with a 23% reduction in the risk of non‐fatal coronary heart disease (Pan et al. 2012). Another clinical study in India showed that α‐linolenic acid ameliorated age‐related neurodegeneration by inhibiting Tau aggregation and modulating Tau conformation (S. E. Desale et al. 2021). These pieces of evidence suggest that α‐linolenic acid is a potential protective factor for longevity. However, this MR analysis reported that sterol ester with α‐linolenic acid (27:1/18:3) was a risk factor for longevity. Therefore, we speculate that the effect of sterol ester (27:1/18:3) in reducing genetic susceptibility to longevity may be mediated by sterol ester and specific fatty acids (27:1) rather than α‐linolenic acid.

Triacylglycerol (52:2) refers to triacylglycerol containing 52 carbon atoms and two double bonds. With aging, the body develops characteristic changes such as increased plasma triacylglycerol levels, decreased postprandial plasma triacylglycerol clearance, decreased lipolysis, and increased ectopic fat deposition, which are closely associated with age‐related metabolic disorders (Spitler and Davies 2020). A 4‐year prospective study from the Research Center Münster, Germany, showed that triacylglycerol levels are associated with the incidence of coronary atherosclerotic disease (Assmann and Schulte 1992). Hypertriglyceridemia is an additional coronary risk factor when triacylglycerol levels and plasma cholesterol content are high (Assmann and Schulte 1992). A Danish prospective study involving 13,956 participants found that the incidence of ischemic stroke increased with increasing levels of triacylglycerol (Freiberg et al. 2008). In a subsequent meta‐analysis, J. Liu et al. (2013) found a dose‐dependent correlation between triacylglycerol levels and the incidence of coronary heart disease as well as all‐cause mortality. They pointed out that for every one millimole per liter increase in triacylglycerol, the risk of cardiovascular disease and all‐cause mortality increased by 13% and 12%, respectively (J. Liu et al. 2013). Another animal study conducted in the United States confirmed that sensible dietary restriction reduced serum triglyceride levels and body fat content in canines, consequently delaying the onset of chronic diseases and significantly extending the median lifespan (Kealy et al. 2002). These pieces of evidence suggest that triacylglycerol reduces longevity by increasing the risk of age‐related metabolic disease, pointing to triacylglycerol (52:2) and triacylglycerol (54:5) as risk factors for longevity.

Phosphatidylcholine (O‐16:0_18:2) refers to phosphatidylcholine with palmitoleic acid (16:0) at the Sn‐1 position and linoleic acid (18:2) at the Sn‐2 position. Phosphatidylcholine, as one of the most abundant phospholipids in cell membranes, is thought to have a positive impact on health (van der Veen et al. 2017). A previous study found that phosphatidylcholine levels are negatively correlated with the rate of biological aging (D. Liu et al. 2023). Additionally, a cross‐sectional study conducted in the UK found significantly reduced levels of phosphatidylcholine in the plasma of 205 AD patients, which supports a positive correlation between plasma phosphatidylcholine levels and cognition within the prefrontal cortex (M. Kim et al. 2017). Another Chinese study showed that dietary supplementation of phosphatidylcholine improved brain learning and memory functions and enhanced resistance to oxidative stress by modulating superoxide dismutase (SOD) activity (Zhou et al. 2016). These pieces of evidence point to phosphatidylcholine possessing antioxidant and anti‐aging potential, suggesting it is a protective factor for longevity.

In addition, palmitoleic and linoleic acids have been recognized as potential protective factors for longevity. Palmitoleic acid is a lipokine that regulates various metabolic processes and exerts a positive effect on diabetes, cardiovascular disease, neurological disease, and cancer (Bermúdez et al. 2022). A Japanese animal experiment demonstrated that palmitoleic acid ameliorated glycemic and dyslipidemia as well as insulin resistance in type 2 diabetic mice by down‐regulating the expression of pro‐inflammatory factors in adipose tissue (Yang et al. 2011). Another American study showed that dietary palmitoleic acid reduced triglyceride levels by 40% and atherosclerotic plaque area by 45% in low‐density lipoprotein (LDL) receptor‐deficient mice (Yang et al. 2019). Additionally, a Chinese study reported that palmitoleic acid dose‐dependently attenuated palmitic acid‐induced microglia damage by inhibiting cellular pyroptosis, apoptosis, and endoplasmic reticulum stress (Yu et al. 2024). These studies support the notion that palmitoleic acid is a protective factor in several diseases. Interestingly, linoleic acid, another free fatty acid of phosphatidylcholine (O‐16:0_18:2), exhibited diametrically opposed effects on organisms at different doses. A French animal experiment found that excessive intake of linoleic acid upregulated the expression of inflammatory factors and increased the risk of atherosclerosis in rats (Manyam et al. 1988). However, another Chinese study showed that 1 µm linoleic acid significantly reduced oxidative stress and prolonged nematode lifespan, whereas 10 µm linoleic acid enhanced oxidative stress and shortened nematode lifespan (T. C. Chen, Hsu, et al. 2023). These pieces of evidence suggest that moderate amounts of linoleic and palmitoleic acids may have a positive effect on longevity, highlighting phosphatidylcholine (O‐16:0_18:2) as a protective factor for longevity.

### 4.3 Effects of Metabolites on Longevity

EDTA is a classical metal chelator that inhibits metal ion–mediated lipid oxidation through chelation reactions, thereby reducing lipid instability and aging processes associated with lipid peroxidation (Lamb and Leake 1992). Metal ion levels in the human body change with age, and modulators of metal homeostasis have been reported to slow down aging by regulating these ion levels (Zeidan et al. 2021). In recent decades, EDTA‐based chelation therapy has been applied to treat vascular diseases, including atherosclerotic plaque blockage. Atherosclerotic plaques are primarily composed of sterol esters and triglycerides and are enriched with iron and copper ions, which catalyze oxidative reactions and can be chelated by EDTA, potentially leading to reduced circulating EDTA levels (Kopriva et al. 2015). The accumulation of iron and copper within arterial plaques, particularly inside macrophages, has been implicated in plaque progression and destabilization via Fenton reaction–mediated oxidative mechanisms (Huang et al. 2025). Conversely, by chelating these metal ions, EDTA may indirectly modulate lipid metabolism by reducing oxidative modifications of LDL cholesterol, thereby influencing the lipid composition and stability of plaques (Xie et al. 2022; McDougall et al. 2019). Compared with statin, EDTA not only prevents the formation and buildup of new plaques but also removes already attached plaques, thereby improving blood circulation (Rowbury 2011). A previous study showed that EDTA reduces the risk of cardiovascular disease by 18%, and this effect is particularly significant in diabetic patients (Avila et al. 2014). Metal chelators, represented by EDTA, have been reported to reduce the risk of cardiovascular disease by 41% and the risk of total death by 43% in diabetic patients (Avila et al. 2014). In addition to cardiovascular disease, EDTA has been used in the treatment of other age‐related diseases such as bone disorders, neurodegenerative diseases, and glaucoma (Ferrero 2022). An Italian study found that EDTA completely eliminated calcifications in 62.5% of patients with shoulder calcific tendonitis and partially reduced them in 22.5% (Cacchio et al. 2009). Another Chinese study found that EDTA‐modified 17β‐estradiol more effectively reversed estrogen deficiency‐induced osteoporosis and reduced the side effects of estrogen‐only therapy (X. Chen et al. 2020). Additionally, when EDTA was combined with permeability enhancers, it inhibited reactive oxygen species production and lipid peroxidation to increase retinal ganglion cell survival and reduce optic nerve demyelination, thereby increasing the improvement of glaucoma (P. Liu et al. 2014). These pieces of evidence suggest that ETDA is associated with delaying aging and promoting longevity.

1‐arachidonoyl‐gpc (20:4n6) is a glycerophosphatidylcholine containing arachidonic acid, composed of 20 carbon atoms and four double bonds on the carbon atom at position six. Glycerophosphatidylcholine is an important phospholipid compound, and its elevation is a significant marker of tumor development (Sonkar et al. 2019). Additionally, as a precursor of arachidonic acid, it is hydrolyzed to arachidonic acid and prostaglandins through a series of metabolic pathways and is involved in inflammatory responses (Astudillo et al. 2021). Arachidonic acid is a polyunsaturated fatty acid esterified in glycerolipids or glycerophospholipids and serves as a precursor of inflammatory mediators with pro‐inflammatory effects (Martin et al. 2016). Inflammation, an endogenous factor that promotes aging, is an important mechanism inducing disease and affecting lifespan (Li et al. 2023). A meta‐analysis involving 54 prospective studies and 160,000 participants revealed that higher levels of inflammatory factors are associated with a relative increase in the incidence and mortality of cardiovascular disease (Emerging Risk Factors Collaboration et al. 2010). A subsequent study found that inhibiting the chronic inflammatory response in aging model mice reduces disease incidence and extends lifespan (Asadi Shahmirzadi et al. 2020). These pieces of evidence suggest that glycerophosphatidylcholine and 2‐arachidonic acid may reduce life expectancy by increasing cancer risk and inducing an inflammatory response, respectively, pointing to 1‐arachidonoyl‐gpc (20:4n6) as a potential risk factor for longevity. However, there is no available evidence regarding potential synergistic effects between EDTA and 1‐arachidonoyl‐gpc (20:4n6) in any disease context. To date, no studies have investigated whether these two metabolites interact to influence lipid metabolism, oxidative stress, or disease progression. Future experimental and clinical studies are warranted to clarify whether EDTA and 1‐arachidonoyl‐gpc have independent or combined effects on metabolic regulation and longevity.

Notably, the above‐mentioned triacylglycerols, sterol esters, and phosphatidylcholine are key lipid metabolites that critically influence inflammation, oxidative stress, and cellular senescence. First, excess triglyceride‐rich lipoproteins induce endoplasmic reticulum and oxidative stress in endothelial cells and macrophages, promoting atherogenesis (Yingchun et al. 2018). Second, oxidized sterol esters exhibit strong cytotoxicity and drive vascular inflammation (Wielkoszyński et al. 2020). Third, oxidative modification of phosphatidylcholine generates bioactive mediators, such as 1‐palmitoyl‐2‐arachidonoyl‐sn‐glycero‐3‐phosphocholine, which critically regulate inflammatory and antioxidant responses in macrophages (Lu et al. 2017). Together, these findings highlight lipid metabolites as central modulators of inflammation, oxidative injury, and aging, providing a mechanistic basis for their role in lifespan regulation.

### 4.4 Effects of Lipidome‐Regulated Metabolites on Longevity

This MR analysis showed that sterol ester (27:1/18:3) and triacylglycerol (52:2) reduced the genetic susceptibility to longevity by decreasing EDTA levels, with EDTA accounting for 13.60% and 11.20% of the reduction in genetic susceptibility to longevity associated with sterol ester (27:1/18:3) and triacylglycerol (52:2), respectively. However, there is currently no direct evidence supporting the relationship of sterol ester or triacylglycerol with EDTA. A previous study showed that iron ion concentration (0.370 nmol/mg vs. 0.022 nmol/mg) and copper ion concentration (2.01 pmol/mg vs. 7.51 pmol/mg) were significantly elevated in atheromatous plaque intima compared to healthy controls, and iron accumulation was positively correlated with cholesterol levels (Stadler et al. 2004). Given that cholesterol and triglycerides are the main substances that make up arterial plaque (Marinello et al. 2003), we hypothesized that the accumulation of iron and copper ions in plaques was associated with elevated cholesterol and triglyceride levels. With the increase in metal ions, EDTA is constantly bound and consumed by metal ions (Zeidan et al. 2021). It in turn leads to a decrease in intracellular EDTA levels and an enhanced oxidative stress response, thereby promoting aging and reduced lifespan (Zeidan et al. 2021).

Additionally, this study also showed that 1‐arachidonoyl‐gpc (20:4n6), as an intermediate metabolite of triacylglycerol (52:4) associated with longevity, accounted for 8.37% of the reduction in genetic susceptibility to longevity associated with triacylglycerol (52:4). This may be because triglyceride‐mediated oxidative stress activates intracellular phospholipase A2 and releases arachidonic acid (Tallima 2021). Subsequently, arachidonic acid exacerbates the inflammatory response through a series of metabolic pathways, ultimately promoting cellular senescence and reduced longevity.

In addition to the above potential risk factors, we found that phosphatidylcholine (O‐16:0_18:2) increased the genetic susceptibility to longevity by increasing EDTA levels, with EDTA accounting for 22.10% of the increase in genetic susceptibility to longevity associated with phosphatidylcholine (O‐16:0_18:2). Given that phosphatidylcholine has a role in regulating SOD activity (Zhou et al. 2016), it may reduce EDTA depletion by inhibiting oxidative stress, thereby enhancing the protective effect of ETDA on cells.

### 4.5 Limitations and Prospects

Although this study provides genetic insights into the analysis of lipidome–metabolites–longevity, there are still several limitations that need to be acknowledged. First, the data on lipidome, metabolites, and longevity included in this study were derived from individuals of European descent, which restricts the generalizability of our findings. Although we attempted to perform similar analyses in Asian and African populations, these analyses could not be conducted due to the lack of publicly available lipidomic and metabolomic GWAS datasets for these populations. Therefore, caution should be exercised when extrapolating our conclusions to non‐European groups, and future studies should aim to collect multiethnic data to validate the associations observed here. Second, the GWAS datasets used in this study did not provide individual‐level information, particularly regarding sex and age. Although we recognize the importance of assessing potential differences across these subgroups, subgroup analyses based on sex‐ or age‐specific longevity data could not be conducted due to the unavailability of corresponding datasets. Future studies with stratified GWAS data are needed to investigate whether the effects of lipidome and metabolites on longevity vary by sex or age. Third, while this study identified four lipidome–metabolite–longevity pathways, there is insufficient clinical evidence to support and explain the associated biological mechanisms. Direct clinical evidence linking these pathways to lifespan extension or aging‐related processes remains lacking.

We expect that future research will continue to improve upon these limitations and make efforts in several directions. First, collecting GWAS data from diverse racial groups and conducting relevant MR analyses will be crucial to evaluate the generalizability of these findings. Second, large‐scale clinical studies should explore the roles of lipidome and metabolites across different sexes and age groups to enrich clinical evidence and strengthen the external validity of the results. Third, animal models could be used to manipulate metabolite levels and assess their effects on aging hallmarks such as telomere shortening, cellular senescence, and oxidative stress. Finally, clinical trials are needed to validate these associations in humans and to explore the therapeutic potential of targeting lipid metabolites for promoting healthy aging.

## 5 Conclusion

The MR analysis revealed four pathways through which lipidome regulates longevity via metabolites. Lipidome such as sterol ester (27:1/18:3), phosphatidylcholine (O‐16:0_18:2), triacylglycerol (52:2), and triacylglycerol (54:5), as well as metabolites such as EDTA and 1‐arachidonoyl‐gpc (20:4n6), may play important roles in longevity. Further studies are needed to explore their roles and mechanisms in regulating longevity in the future.

## Author Contributions

**Yunfeng Yu**: writing – original draft, conceptualization, supervision. **Xinyu Yang**: writing – original draft, methodology, data curation. **Juan Deng**: writing – original draft, methodology, formal analysis. **Jingyi Wu**: writing – original draft, methodology. **Rong Yu**: writing – review and editing, supervision. **Qin Xiang**: writing – review and editing, formal analysis, supervision. All authors participated in the revision of the manuscript, read and approved the submitted version.

## Funding

This study was supported by the Hunan University of Chinese Medicine Disciplinary Construction' Revealing the List and Appointing Leaders' Project (22JBZ002).

## Ethics Statement

This study is based on published experimental research and is not currently applicable to medical ethics.

## Conflicts of Interest

The authors declare no conflicts of interest.

## Supporting information




**Supporting Fig. 1**: brb370937‐sup‐0001‐FigureS1.jpg

**Supporting Fig. 2**: brb370937‐sup‐0002‐FigureS2.jpg

**Supporting Fig. 3**: brb370937‐sup‐0003‐FigureS3.jpg

**Supporting Tables**: brb370937‐sup‐0004‐TableS1‐S3.xlsx

## Data Availability

Original contributions from this study are included in the article/Supporting Information. For further inquiries, please contact the corresponding author.
