# Supplementary figures and images for: Longevity Code: Lipidome Associations and Mediated Metabolite Effects
Source: Brain Behav. 2025 Nov 11;15(11):e70937. doi: 10.1002/brb3.70937 (PMC12611319; doi:10.1002/brb3.70937)

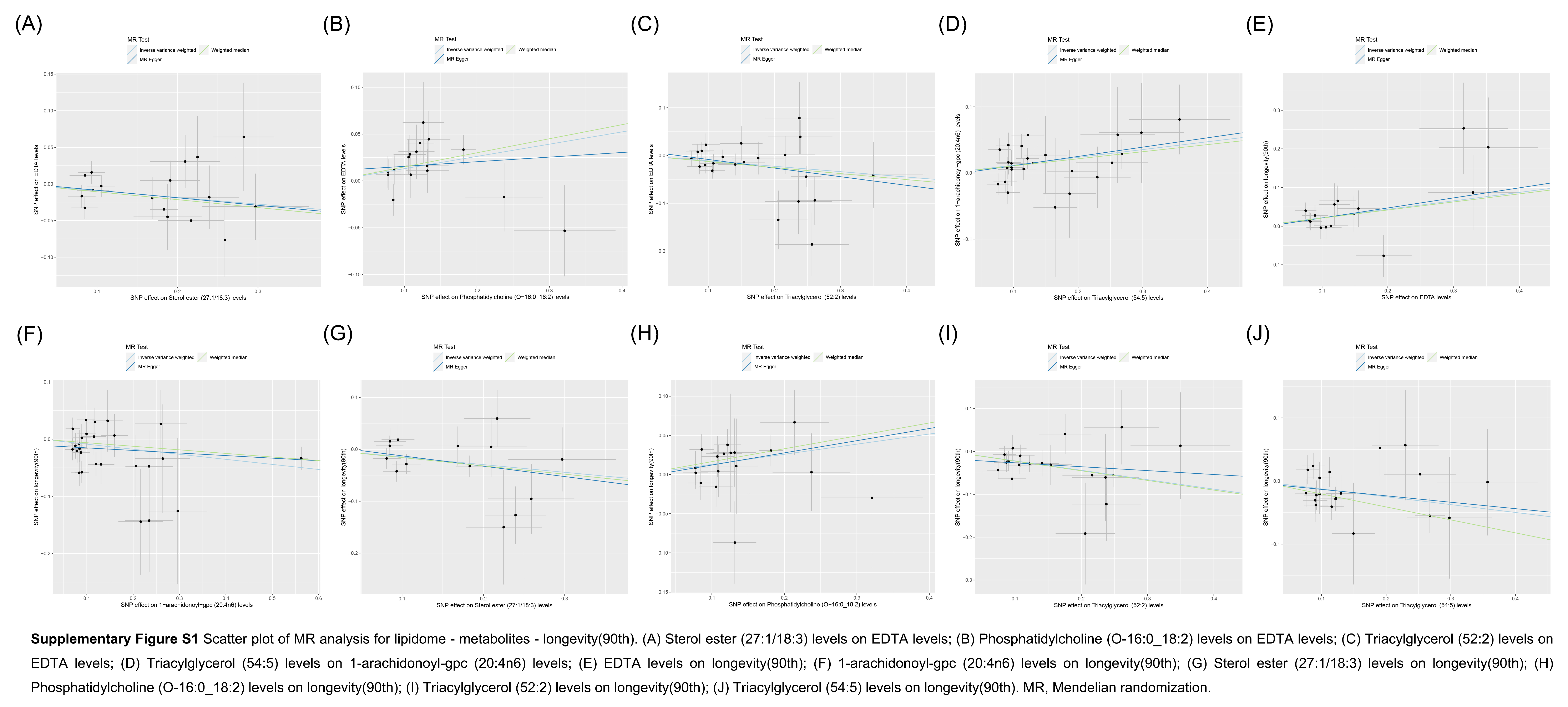

Supplement: Supplementary file 1 — Supporting Fig. 1: brb370937‐sup‐0001‐FigureS1.jpg [file BRB3-15-e70937-s001.jpg]

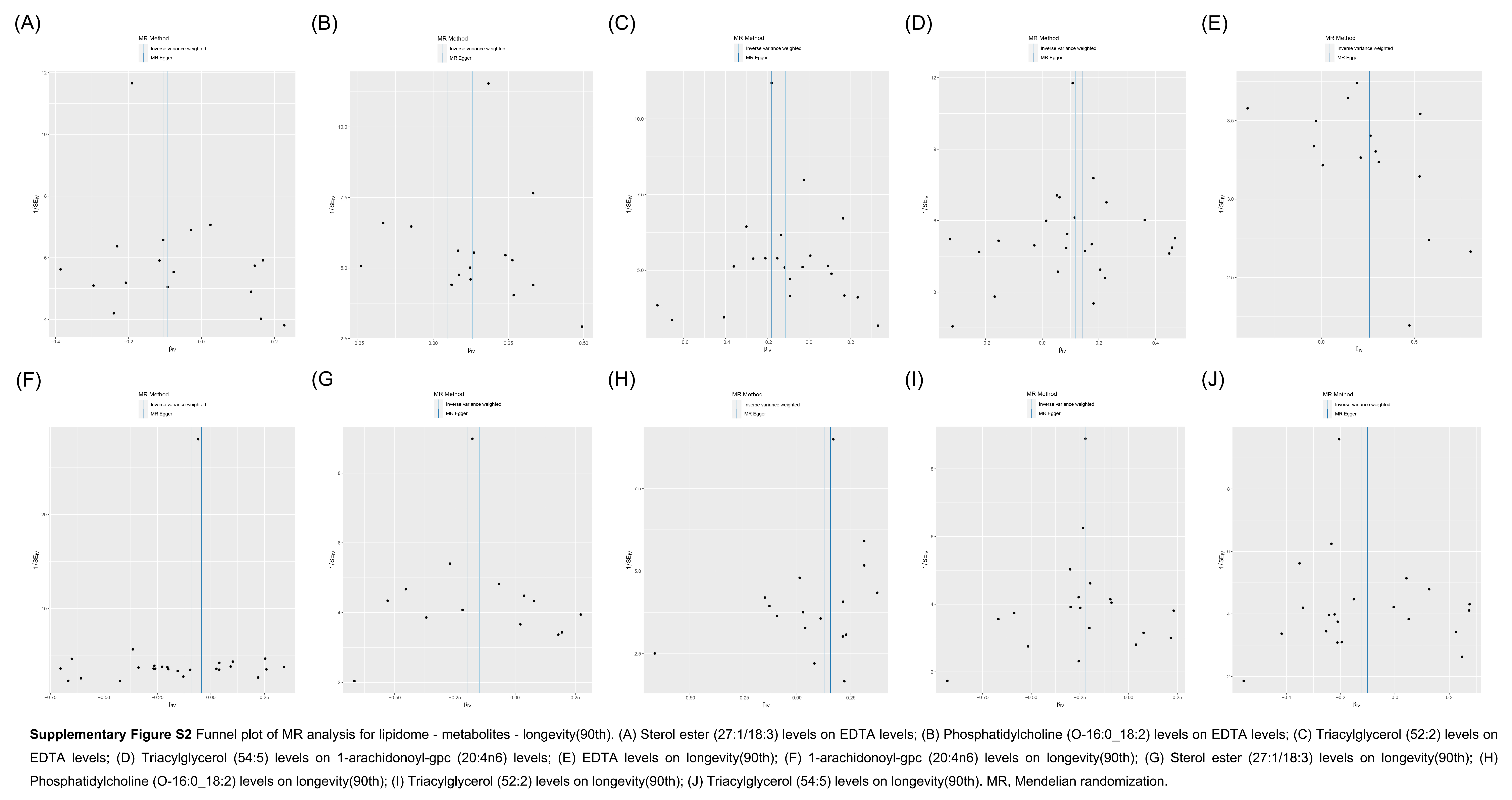

Supplement: Supplementary file 2 — Supporting Fig. 2: brb370937‐sup‐0002‐FigureS2.jpg [file BRB3-15-e70937-s002.jpg]

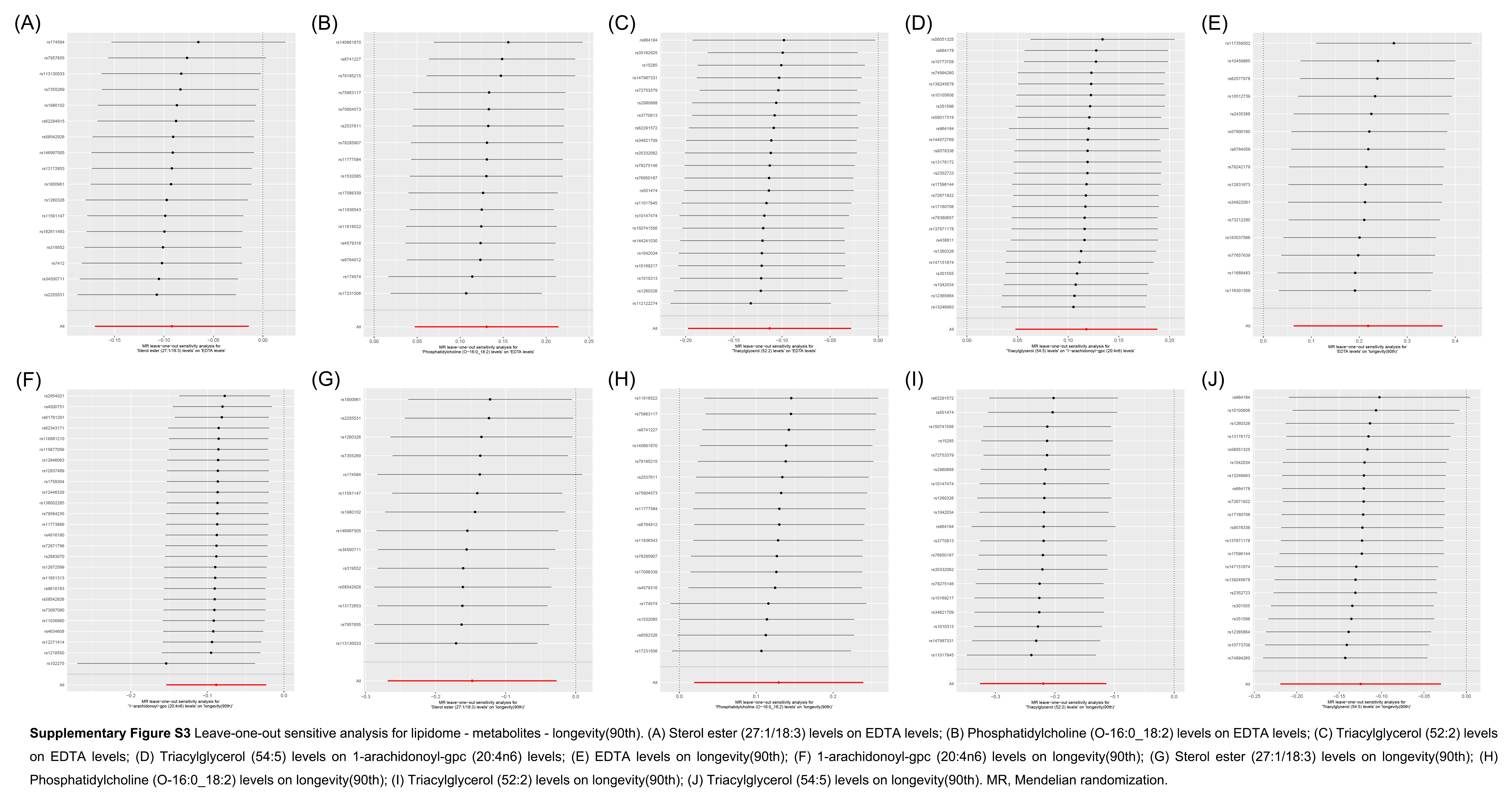

Supplement: Supplementary file 3 — Supporting Fig. 3: brb370937‐sup‐0003‐FigureS3.jpg [file BRB3-15-e70937-s004.jpg]
